# Anæsthesia and Anæsthetics.—Criticism of an Address by Dr. Jno. J. Caldwell

**Published:** 1879-07

**Authors:** Martin P. Scott


### ARTICLE II.

(A Review and Criticism on the Transactions of the Dental Association of Maryland and District of Columbia.)

### *Anæsthesia and Anæsthetics.*

ADDRESS BY DR. JOHN J. CALDWELL, BALTIMORE.

I have read with much pleasure Dr. Caldwell's address, and received much instruction. It is a well considered and thoughtful resume of the subject of anæsthetics to the present time. Not only has he presented the views of the ablest writers on the subject, but has added his own comments on their physiological action, which certainly are worthy to rank with the best. Dr. C justly gives the first rank to chloroform in all operations, except those in dental surgery, which last requires an erect or partly erect posture. In such cases nitrous oxide gas is preferable. Chloroform is better than ether or any of its mixtures; chloroform is King. In the chair of criticism, I avail myself of the critic's privilege and speak "*ex cathedra.*"

The mode of administration is dwelt upon at sufficient length, where he says the patient must be recumbent, the stomach empty and sufficient amount of atmospheric air admitted along with the agent. I am sorry he did not condemn in stronger language the pernicious and dangerous practice of over-powering the patient at once with a large quantity of chloroform; better garotte to insensibility and proceed with the operation.*

The precautions against swallowing the tongue (a kind of felo-de-se,) are made sufficiently prominent, as well as the

---

[*The practice of rapid administration and quick overwhelming of the power of resistance finds many advocates among the best men—indeed the leaders—at this day.—*Eds. Amer. Jour. of Dental Science.*]

physiological condition upon which the accident supervenes. It is regretted that he did not analyze with sufficient exhaustiveness the causes of death, as by coma, asphyxia and the like, as in that event he would have traced with but few exceptions death to failure to admit atmospheric air in sufficient quantities.

I have administered chloroform many hundred times, have witnessed its administration in the hospitals of Paris, in the field and in hospitals during the late civil war, and have yet to see the least unpleasantness follow where this precaution has been used.

Two points should be borne in mind,—the carbonized condition of the blood, and the specific action of the agent upon the brain and other nerve centres. People vary in susceptibility to its influence. The negro succumbs more readily than the white race, a physiological fact to which I would invite the attention of those who insist upon negro equality.

I take this occasion to say that the views of Dr. Chisholm in reference to the necessity of bringing the patient fully under the influence of chloroform before commencing an operation, to render the reflex motor system insensible as well as the sentient nervous centres, have my hearty concurrence. Shock is thus avoided, which we know is so often fatal.

Chloroform has not been considered by the Doctor, or by any of the authors cited, as a therapeuetic agent in the treatment of disease. A substance which acts so powerfully upon every part of the nervous system, centricly and excentric, must be a most important remedial agent in a large number of diseases, for there is scarcely one in which the nervous system is not more or less concerned. As early as 1856 I used it in treatment of colic with magical effect. During the late war colic was very common among our soldiers returned from Northern prisons. I used it in a number of severe cases and always with most satisfactory results. It does more than temporary relieve pain, for in many cases the pain does not recur after the efforts of the chloroform have apparently subsided. In passage of calculus from kidney, it is emphatically *the remedy*, at least in my hands I have had but little trouble with this affection. I administer by inhalation. I have used it but once only in the cold stage of intermittent. The patient had been shaking for two hours in a way I never saw any one else shake, black under the eyes—an Arkansas shake. I thought she would die. I gave chloroform by inhalation, when the paroxism ceased instantly and did not return. A slight fever followed. I used it on the same person a second time under the same circumstances. On this occasion the shivering and shaking returned. I repeated chloroform with arrest and recurrence of symptoms. At the end of about twenty minutes, being under its influence from time to time, the cold stage was arrested. I would suggest the use of chloroform in intermittent and malarial fevers in their outset as a curative agent. Would that some substitute might be discovered for quinine to deprive those harpies of Philadelphia of their blood money, wrung from the sick poor of our country through the instrumentality of an iniquitous tariff.

Chloroform is worthy of a trial in that scourge of the tropics and our Southern Country—Yellow Fever. I suggest it in these cases upon the idea of the same theory of such diseases, the most rational yet put forth of their pathology.

I trust that Dr. Caldwell at some future time will discuss the use of chloroform applied to treatment of diseases. Internally I would suggest it as a remedy for all parasites, it will kill the "bott," so fatal to the horse.

In that part of the address under criticism in which the methods of resuscitation from the effects of chloroform are discussed, prominence is given to the use of Nitrite of Amyl. (See Dabney's writings in which interesting experiments are cited upon dogs, showing its powerful effects, due as he believes to its action on the heart, conclusions which are strengthened by two cases of resuscitation reported by Dr.

R. W. Nelson, of Virginia.) It is worthy of remark that of the four dogs experimented upon, the only one not revived by the Nitrite of Amyl, was the one to whom chloroform had been given *unmixed with atmospheric air.*

Artificial respiration is also recommended. Dr. Cutter, of Boston, has furnished what has been so much needed, a simple mechanical contrivance to pump air or oxygen into the lungs and pump it out. "To pump air in and out of the body without any pressure on the chest—to breathe life into persons."

But I would ask especial attention to that part of Dr. Caldwell's essay which treats of the application of Faradaiac Electricity. Some of the experiments cited upon the pneumogastic nerve after its division, in which electricity supplied the place of nerve force in digestion, are most interesting. It has long been known that electricity is a powerful nervous stimulant, but here we have the fact arrived at of idenity of nerve power and electric force.* The process of digestion being carried on by substituting a battery for the nerve centres of the pneumogastric nerves.

Such being the case, there cannot rest a doubt that in the battery we have the most powerful and efficient means of restoring persons to life when every other known agent would fail. As merely an aid, a stimulant, its powerful effects are recognized, but if indeed the subtle agent we call electricity be the same as nerve force we shall have attained a point in physiology which may enable us to unravel the problem of life. Setting aside all that our confrere has said in regard to chloroform, its physiological action, its mode of administration &c., the means of restoration from

---

[*This does not follow necessarily. Although "like causes produce like results," is an axiom, yet in this case neither the cause nor effect in the one case or the other, *i. e.*—the relation between mere force and identity and their coincidence in effect on the pneumogastric are fairly understood. We do not think Dr. C., could fairly be construed as claiming to establishéd complete identity, but only great similarity.—*Eds. Amer. Jour. of Dental Science.*]

its pernicious effects, I regard his suggestion of electricity as the most potent remedy, and opening a new field for its application as a therapeutic agent in the treatment of disease, and trust that Dr. Caldwell will again give public the benefit of his experience and reflection upon Faradaiac Electricity.

In conclusion I would recommend all who desire in brief to review the subject of anæsthetics to consult Dr. Caldwell's admirable resume of the subject as set forth in the transactions of the Dental Association of the State of Maryland and District of Columbia for the year 1878.

I cannot close my remarks without a word to the Dental Profession. I am happy to congratulate them upon the great advance and upward progress of the profession of dentistry. Like surgery, it long remained a purely mechanical art. Time was when surgery was a part of the barber's calling. Time was when medicine was so low that the priests were inhibited from its practice, although in England, Macauley tells us they (the priests) sometimes curried the coach horses and were prohibited from marrying the servant girls. The progress of intellectual development has taken all these high callings from their menial condition. The priest exercises the noblest profession of the world; he is the minister and interpreter of God's holy word, next to his is the mission of those who minister to the sick in body. Dentistry must now takes its place as a branch of surgery; one of her schools has set us a noble example by which great reforms may be effected in medical education, so much needed in these days of medical multiplication. That proficiency, not length of time of study, should be the test demanded for graduation, and that the teachers should never be permitted to examine candidates or grant diplomas.

MARTIN P. SCOTT, M. D.

*No.* 68 *W. Fayette Street, Baltimore, Md.*

